# Editorial: Exploring Plant Rhizosphere, Phyllosphere and Endosphere Microbial Communities to Improve the Management of Polluted Sites

**DOI:** 10.3389/fmicb.2021.763566

**Published:** 2021-10-06

**Authors:** Michel Chalot, Markus Puschenreiter

**Affiliations:** ^1^UMR Chrono-environnement, CNRS 6249 - Université de Bourgogne-Franche-Comté, Besançon, France; ^2^Université de Lorraine, Faculté des Sciences et Technologies, Nancy, France; ^3^University of Natural Resources and Life Sciences Vienna, Vienna, Austria

**Keywords:** microbial communities, polluted sites, plant growth promoting microorganisms (PGPM), high-throughput sequencing technologies, plant inoculation

Soils are an important and non-renewable resource and fulfill several crucial ecological functions, including plant production, nutrient cycling, carbon storage, water maintenance, and many more (Hatfield et al., 2017). Soils are, however, also subject to pollution by various chemicals, such as trace metals, petroleum hydrocarbons, and xenobiotics. Plant microbial communities are inherent to plants' adaptation to their changing environment, while plants provide organic fuels for microbial functioning in the rhizosphere. Plant growth promoting microorganisms (PGPM), such as plant growth promoting bacteria (PGPB), ectomycorrhizal and endomycorrhizal fungi (EMF and AMF) are key players in this context and have been shown to promote pollutant degradation or containment in plant leaves or roots (Kidd et al., 2015; Phanthavongsa et al., 2017; Riaz et al., 2020) while improving plant growth (Ma et al., 2011; Cundy et al., 2016; Ciadamidaro et al., 2017). In the context of phytoextraction PGPM may also increase contaminant uptake and accumulation. Various studies have reported a high diversity of microbes in the rhizosphere and endosphere of metal accumulating plants (Thijs et al., 2017). New developments of “omics” tools are increasingly contributing to the understanding of the mechanisms and processes involved in plant-microbe interactions. However, these intimate relationships may be profoundly modified in polluted environments, and using PGPM in these degraded environments as nature-based solutions is a real, but promising challenge. The goal of this Research Topic is to explore and better understand the complex interactions between pollutants, soil, plant roots and leaves, and microorganisms, providing an appropriate platform to disseminate recent results in this research area. PGPM are considered as environmentally friendly and efficient microbes. However, the potential of emerging microbes such as phyllosphere yeasts or dark septate endophytes remain to be explored. It is also crucial to elucidate whether the microbial machineries that allow microbes to degrade or contain pollutants are fully expressed under interactions with their plant host, and how the processes involved may be better exploited in the revitalization or remediation of polluted soils.

This Research Topic of *Frontiers in Microbiology*, “*Exploring Plant Rhizosphere, Phyllosphere and Endosphere Microbial Communities to Improve the Management of Polluted Sites*,” presents 21 original research articles that span the large field of research on polluted soils and give new insights based on novel results, thus providing a basis for further studies and potential application options. The retrospective analysis of the articles contributed to this RT allowed them to be assigned to one of 2 main sub-topics (i) the development of next generation sequencing (NGS) for the characterization of microbial communities in contaminated soils using massively parallel sequencing technology that offers ultra-high throughput, scalability, and speed; (ii) the selection of microbes for the inoculation of suitable plants in remediation approaches.

## High-Throughput Sequencing Technologies for the Characterization of Microbial Communities

High-throughput sequencing technologies have been used to uncover the structure and composition of microbial communities in the rhizosphere and endosphere of tree roots in contaminated soils and these technologies have been widely employed in various pollution contexts. Within this topic, pollution with organic and metal compounds have been addressed.

Regarding pollutions with organic compounds, Wang Q. et al. explored the assemblage of soil microbial communities and their pollutant-degrading potential in soil undergoing a bioremediation process termed Ecopiling, which involves biostimulation of indigenous hydrocarbon degraders. They observed the presence of less abundant genera of bacteria such as *Pusillimonas, Dietzia*, and *Bradyrhizobium*, which metagenomes exhibited hydrocarbon degradation genes and which represent relevant targets for the development of bioaugmentation strategies of hydrocarbon-contaminated soil. A key point raised by Barra Carrociolo et al. is that we lack long-term studies in field settings. They studied the natural microbial abundance and the composition of the bacterial community using NGS in a plant-assisted bioremediation strategy at PCB-contaminated sites. They found higher percentages of bacteria genera (i.e., *Stenotrophomonas, Pseudomonas, Rhizobium, Skermanella*) typically involved in xenobiotic degradation in the rhizosphere, demonstrating the effectiveness of the poplar in promoting microbial transformations.

Beyond organic pollution, metals are well-known significant pollutants in soils (Alloway, 2013). In their contribution, Shi Q. et al. investigated the bacterial community structure in the rhizosphere of an Al-tolerant and an Al-sensitive wild soybeans exposed to aluminum. Their results pointed out that the high concentration of Al has increased the difference in rhizobacterial community structure between the two genotypes. Raveau et al. also addressed the question of microbial communities in a cultivated plant by studying the root bacterial communities of the aromatic plant, clary sage, cultivated on the aged TE-contaminated soils, as a first step in improving microbial-based phytomanagement approaches. The bacterial genera *Pseudarthrobacter, Streptomyces*, and *Actinoplanes* were highly abundant in the rhizosphere of clary sage and may represent relevant microbial targets. Martos et al. further explored the potential role of microbiota to the hyperaccumulation phenotype of *Noccaea brachypetala*. Putting forward the hypothesis that the microbiome companion of the plant roots may influence the ability of these plants to take up metals, they employed high-throughput sequencing of the bacterial and fungal communities in the rhizosphere soil and rhizoplane fractions and highlighted out an enrichment in the hyperaccumulator rhizoplane of metal-tolerant bacteria and bacteria involved in nitrogen cycling. In an effort to clarify the influence of plant and soil traits on fungal community structure during plant development at copper tailings dam, Jia et al. 1,818 total views investigated dynamic changes in rhizospheric and phyllospheric fungal communities during different plant development stages. They found that phyllosphere and rhizosphere fungal communities possessed distinct functional features, clarifying the dynamical relationships between phyllosphere and rhizosphere fungal communities.

## Selection of Microbes for the Inoculation of Suitable Plants

Microorganisms typically influence significantly plant-soil interactions as well as soil remediation processes. These microorganisms often include native populations, but may also be inoculated to the polluted soil. A general overview on the role of soil bacteria in remediation processes is given by González Henao and Ghneim-Herrera. Regarding metal pollutants, microorganisms may increase the metal uptake by plants and thus improve the phytoextraction efficiency. Yung et al. reported that fungal endophytes enhanced the Zn accumulation by the hyperaccumulator *Noccaea caerulescens*. The Zn phytoextraction capacity was also enhanced by the increase of plant biomass. The latter was likely related to enhanced nutrient supply and thus improved plant nutrition.

Soil microorganisms may also increase plant growth and/or metal tolerance of plants. A comprehensive overview on the effect of endophytic bacteria on the latter aspect is given in the meta-analysis by Franco-Franklin et al. More specific, Niu et al. found that inoculating soil with *Bacillus* spp. and *Aspergillus* spp. enhanced growth and metal accumulation in a *Salix* species, but the relative increase of both biomass and pollutant concentrations remained on a moderate level of 10–15%. Not only trees, but also annual or perennial plants that grow fast and produce huge biomass in a short time period are often used in phytoremediation approaches. In this context, Ferrarini et al. found that inoculated bacteria in combination with a chelating agent (EDTA) enhanced the metal accumulation in giant reed and hemp plants. Soil microorganisms may also alleviate potential toxicity effects, as reported by Zhang et al.: they found that *Rhizobia intraradices* inoculated to *Robinia pseudoacacia* reduces As toxicity effects.

Concerning organic pollutants, soil microorganisms are typically key players in degradation processes. Inoculating polluted soil may increase the degradation processes and thereby reduce the time needed for remediation. This observation was reported by Zuzolo et al., who found enhanced degradation of total petroleum hydrocarbons by adapted microorganisms in the rhizosphere of two grass and two legume species. The rhizospheric microbes, however, also contributed to improved plant growth and alleviated contaminant-induced stress in plants.

## Conclusions

More detailed insights into microbial community dynamics in polluted soils are a major achievement in recent research activities, which were benefitting from novel methodological advancements, such as high-throughput sequencing technologies. Concerning the effect of microbes on pollutant degradation, immobilization or plant uptake, the effectiveness was confirmed. Further improvements by e.g., selecting more efficient microbes should be the focus of future investigations. More importantly, the use of microbial consortia presents a new approach for efficient remediation of complex mixtures of contaminants and a viable option to share the undesired metabolic burden among various microbial strains. However, many uncertain factors, such as the little elucidated mechanism of cell-to-cell communications, the change in the structure of the microbial population, and the compromise of culture conditions, still exist when consortia of microorganisms are used. To solve these problems, in-depth studies focusing on the interaction mechanisms of natural microbial consortia, the introduction of functional support materials and the development of analytical and predictable computer models should be carried out in the future.

## Author Contributions

MC and MP contributed to the writing of this editorial and approved its content. Both authors contributed to the article and approved the submitted version.

## Funding

MC was supported by the French ANR. MP was supported by the Federal Ministry of Agriculture, Regions and Tourism, Vienna, Austria. MC and MP received funding from the European Union's Horizon 2020 research and innovation programme (project NETFIB). The project NETFIB was carried out under the ERA-NET Cofund SusCrop (Grant No. 771134), being part of the Joint Programming Initiative on Agriculture, Food Security and Climate Change (FACCE-JPI).

## Conflict of Interest

The authors declare that the research was conducted in the absence of any commercial or financial relationships that could be construed as a potential conflict of interest.

## Publisher's Note

All claims expressed in this article are solely those of the authors and do not necessarily represent those of their affiliated organizations, or those of the publisher, the editors and the reviewers. Any product that may be evaluated in this article, or claim that may be made by its manufacturer, is not guaranteed or endorsed by the publisher.
